# Draft Genome Sequence of *Pseudomonas syringae* pv. syringae ALF3 Isolated from Alfalfa

**DOI:** 10.1128/genomeA.01722-15

**Published:** 2016-02-11

**Authors:** James Harrison, Melinda R. Dornbusch, Deborah Samac, David J. Studholme

**Affiliations:** a Biosciences, University of Exeter, Devon, United Kingdom; b USDA-ARS, Plant Science Research Unit, St. Paul, Minnesota, USA; c Department of Plant Pathology, University of Minnesota, St. Paul, Minnesota, USA

## Abstract

We report here the annotated draft genome sequence of *Pseudomonas syringae* pv. syringae strain ALF3, isolated in Wyoming. A comparison of this genome sequence with those of closely related strains of *P. syringae* adapted to other hosts will facilitate research into interactions between this pathogen and alfalfa.

## GENOME ANNOUNCEMENT

*Pseudomonas syringae* pv. syringae causes bacterial stem blight of alfalfa (*Medicago sativa*, also known as lucerne), the most widely cultivated perennial forage crop worldwide (1). The disease is widespread in the central and western United States and occasionally occurs in eastern states. It has also been reported in Australia and Europe, and it was recently reported in western Iran (2); however, little is known about the pathogen. The bacterium penetrates host stems primarily at frost injury sites and forms water-soaked lesions that extend down the stem, becoming amber with dried bacterial exudate that blackens with age. Leaves become water soaked and then chlorotic and necrotic. Plants with the disease are stunted, with spindly stems that are easily broken. Strain ALF3 was isolated from alfalfa plants with symptoms of bacterial stem blight from an area near Cheyenne, WY, and shown to be highly pathogenic on alfalfa, *Medicago truncatula*, and snap bean seed pods (3).

We used the Illumina HiSeq 2500 to generate 13 million pairs of 100-bp reads from genomic DNA; the raw data are available in the Sequence Read Archive (4). *De novo* assembly with Velvet 1.2.03 (5) resulted in 29 scaffolds comprising a total of 130 contigs. The contig *N*_50_ and scaffold *N*_50_ were 127,021 bp and 500,801 bp, respectively. The genome sequence was annotated using the NCBI Prokaryotic Genome Annotation Pipeline (6) version 2.6 (rev. 440435). This predicted 4,842 protein-coding genes and 38 pseudogenes, as well as 46 tRNA genes and three rRNA genes (5S, 16S, and 23S). Intriguingly, the genome sequence of ALF3 is almost identical to that of *P. syringae* B576 (GenBank accession no. JRUO00000000.1), with which it shares 99.9% average nucleotide identity (ANI) over 99.9% of the genome. Little information is available for strain B756 other than that it was isolated from a nonspecified plant in western China. Comparisons of whole-genome sequences revealed that apart from strain B756, strain ALF3 is most similar to *P. syringae* pv. japonica M301072 from barley and *P. syringae* pv. aptata DSM 50252 (7) from beet, sharing 98.7% ANI with each. However, approximately 6% of the ALF3 genome shares no detectable nucleotide sequence similarity with genome sequences of M301072 and DSM 50252. This portion includes putative phage-associated genes and a gene encoding an HrpZ harpin (GenBank accession no. KFF85522.1).

The availability of this genome sequence will enable studies on the molecular basis for adaptation to alfalfa, facilitated by the availability of sequences from other *P. syringae* pv. syringae strains not known to infect this host (7–12) and by the availability of genome sequences from the pathogen responsible for the other major bacterial disease of alfalfa, namely, *Xanthomonas alfalfae* subsp. *alfalfae,* which causes bacterial leaf spot (13).

### Nucleotide sequence accession numbers.

This whole-genome shotgun project has been deposited in DDBJ/ENA/GenBank under the accession no. JPNN00000000. The version described in this paper is the first version, JPNN01000000.
